# Interpretable machine learning for early detection of carbapenem-resistant *Klebsiella pneumoniae* in ICUs: risk prediction using LASSO and XGBoost

**DOI:** 10.3389/fpubh.2026.1798927

**Published:** 2026-04-22

**Authors:** Qiuyan Pan, Xiaoyu Zhou, Biying Zhang, Jiazheng Li, Zhuo Wang, Yong Guo

**Affiliations:** Department of Clinical Laboratory, Suzhou TCM Hospital Affiliated to Nanjing University of Chinese Medicine, Suzhou, Jiangsu, China

**Keywords:** Carbapenem resistance, intensive care unit, *Klebsiella pneumoniae*, LASSO, SHAP, XGBoost

## Abstract

**Background:**

Carbapenem-resistant *Klebsiella pneumoniae* (CRKP) infections in intensive care units are associated with poor outcomes. The delay in obtaining culture-based susceptibility results often forces clinicians to choose between under-treatment and overtreatment with empirical antibiotics. A reliable early risk assessment using only standard clinical data could help address this challenge.

**Methods:**

This single-center retrospective cohort study included 401 ICU patients with culture-confirmed *K. pneumoniae* infections (January 2022 to January 2025). Patients were randomly allocated to training (*n* = 281) and validation (*n* = 120) sets. Predictors extracted from electronic health records comprised demographics, severity scores (APACHE II, SOFA), comorbidities, invasive procedures, inflammatory markers, specimen type, history of multidrug-resistant (MDR) infection, and antibiotic exposure within the preceding 90 days. Feature selection was performed using Least Absolute Shrinkage and Selection Operator (LASSO) regression on the training set. The selected features were used to develop an XGBoost model, whose performance was compared against six other machine learning algorithms (logistic regression, random forest, etc.). Model discrimination was evaluated using the area under the receiver operating characteristic curve (AUC), calibration with Brier scores and calibration curves, and clinical utility with decision curve analysis. SHapley Additive exPlanations (SHAP) values were employed to interpret the model.

**Results:**

CRKP isolates accounted for 15.7% (63/401) of cases. LASSO regression identified nine predictors: procalcitonin (PCT), specimen type, prior MDR infection, prior carbapenem exposure, history of stroke, APACHE II score, white blood cell count, age, and hemoglobin. In the independent validation set, the XGBoost model achieved an AUC of 0.852 (95% CI: 0.745–0.959), with a sensitivity of 0.737, specificity of 0.891, accuracy of 0.867, and an F1-score of 0.636. The model demonstrated good calibration (Brier score: 0.088) and provided a net clinical benefit across a wide range of risk thresholds. SHAP analysis highlighted PCT, specimen source (blood), and prior resistance-related exposures as the most influential predictors.

**Conclusion:**

The integration of LASSO feature selection with the XGBoost algorithm, utilizing only routine clinical data, generates a reliable early-warning model for CRKP infection risk prior to the availability of susceptibility reports. This tool shows promising discriminative ability and calibration, offering potential to guide empirical therapy and support antimicrobial stewardship. Future multicenter prospective studies are warranted to validate its generalizability and real-world clinical impact.

## 1 Introduction

*Klebsiella pneumoniae* is a leading cause of healthcare-associated infections, with particularly severe consequences in critical care settings (1). The rising incidence of carbapenem-resistant *K. pneumoniae* (CRKP) poses a formidable challenge, fueled by extensive carbapenem use and a slow pipeline of novel antimicrobial agents. Globally, CRKP accounts for approximately 60% of carbapenem-resistant Enterobacterales (2). In China the climb has been steep—from 2.4% in 2005 to 13.4% by 2014 (3)—and recent CHINET surveillance puts meropenem resistance at 23.6% and imipenem at 22.5% for 2023 (4). Mortality with CRKP consistently runs 40–50%, far higher than with susceptible strains (5).

The real difficulty lies in the wait for definitive results. Culture and susceptibility testing usually take at least 24–48 h (6, 7). Until then, clinicians must decide whether to include agents such as tigecycline or colistin—both toxic and prone to breed further resistance (8, 9)—or risk inadequate coverage that can prove fatal in septic shock (10, 11). Consequently, a reliable method to stratify patient risk at the time of specimen collection could significantly improve clinical decision-making (12).

Earlier risk models have often been too narrowly focused to travel well. Pediatric nomograms, for example, reflect infection patterns and prescribing habits quite different from adult ICUs (13). Efforts confined to neuro-ICUs (14) or cerebrovascular units (15) are informative but hard to extend. While models derived from broader hospitalized cohorts (16) are more inclusive, they may lack precision when applied to the critically ill due to heterogeneity in illness severity and treatment exposures.

To address this gap, we aimed to develop and validate a pragmatic prediction model specifically for adult ICU patients. Recent advances in machine learning have enabled early prediction of various intensive care unit outcomes using routinely collected intensive care unit data. For example, Shan et al. (17) developed an XGBoost-based model using data from the MIMIC-IV database to predict the incidence of sepsis in intensive care unit patients. Our objective was to utilize exclusively routine clinical and laboratory data, with no requirement for specialized tests, thereby ensuring immediate clinical applicability. We explicitly incorporated detailed antibiotic exposure history and prior MDR infection status as key candidate predictors. A machine learning framework integrating LASSO for feature selection and XGBoost for modeling was employed and compared against traditional and other machine learning methods. Furthermore, we provided an interpretable nomogram and used SHAP analysis to elucidate the contribution of individual predictors, enhancing the model’s transparency and potential for clinical adoption.

## 2 Methods

### 2.1 Study design and ethics

This retrospective cohort study was designed to develop and validate a prediction model for CRKP infection at the time of specimen collection, before antimicrobial susceptibility results were available. The study was conducted at a single academic medical center and reported following the Transparent Reporting of a multivariable prediction model for Individual Prognosis Or Diagnosis (TRIPOD) guidelines (18). The study protocol was approved by the Institutional Review Board of Suzhou TCM Hospital Affiliated to Nanjing University of Chinese Medicine. The requirement for informed consent was waived due to the retrospective nature of the study, which involved the analysis of fully anonymized patient data. This study was conducted in accordance with the Declaration of Helsinki and all applicable ethical standards for research involving human subjects.

### 2.2 Study population

We reviewed all ICU stays at our hospital between January 2022 and January 2025.

Patients were eligible if they: (1) stayed ≥24 h in the ICU; (2) had a clinical specimen collected within 48 h of ICU admission that subsequently grew *Klebsiella pneumoniae*; and (3) had complete medical records available. The index date (time zero) was defined as the date and time of collection of this first *K. pneumoniae*-positive specimen during the ICU stay. All predictor variables were measured using data available prior to or at this time point, ensuring the model’s applicability.

We excluded anyone with >20% missing key variables, known CRKP carriage or infection before admission, or subsequent episodes (only the first positive culture counted).

**Figure 1:**
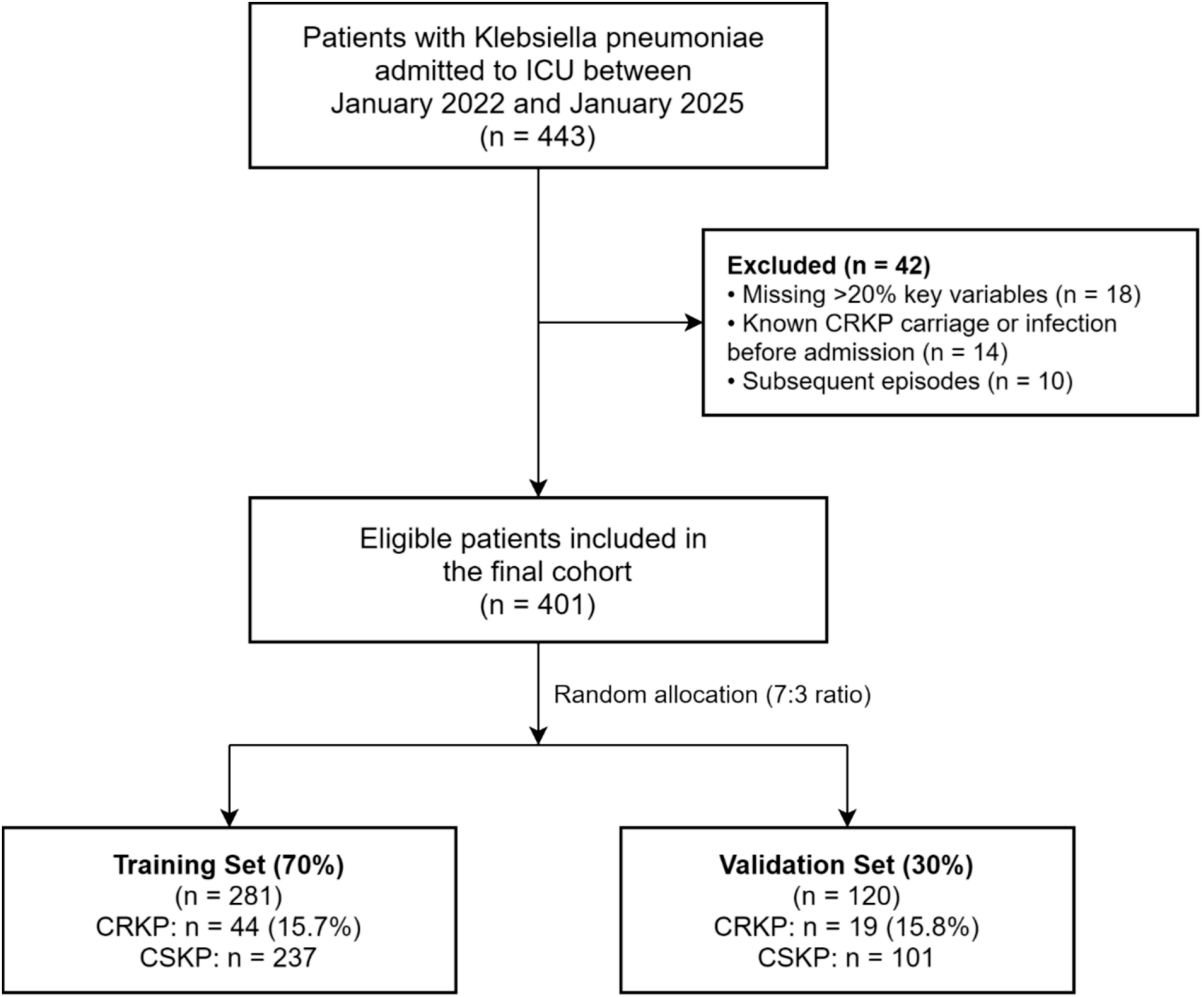
Flowchart of the patient selection and enrollment process.

A total of 401 patients were ultimately included and randomly assigned to the training and validation sets in a 7:3 ratio using random number allocation (Figure 1).

### 2.3 Data collection and variable definitions

Variables came from seven routine categories in the electronic system:

Demographics (age, sex, BMI).

Admission severity scores (APACHE II, SOFA) (19).

Comorbidities (hypertension, diabetes, coronary disease, chronic kidney disease, malignancy, COPD, immunosuppression, etc.)

Invasive procedures (days on mechanical ventilation, central lines, nasogastric tubes, drains).

Antibiotics given in the 90 days before the index specimen (carbapenems, cephalosporins, fluoroquinolones, penicillins, aminoglycosides). This 90-day window was chosen based on the IDSA 2024 guidance, which recommends considering antibiotic exposure within the preceding 3 months for empirical therapy decisions (20). The same window was applied uniformly to all antibiotic classes to maintain consistency. However, the sources of antibiotic exposure data collection are heterogeneous, including inpatient prescriptions, clinical medical records (with accurate dates), and outpatient records (usually recorded only as qualitative descriptions, but without accurate dates). This heterogeneity makes it impractical to systematically extract and standardize antibiotic exposure time in all patients, so we cannot further stratify exposure based on recent (e.g.,≤30 days) or long-term exposure.

Any MDR infection or colonization in the 3 months before admission.

Laboratory results (white blood cells, hemoglobin, platelets, creatinine, procalcitonin, C-reactive protein, albumin).

CRKP was defined by resistance to imipenem, meropenem, or ertapenem according to CLSI M100 guidelines; carbapenem-susceptible isolates had to be sensitive to all three (21). Carbapenem-susceptible *K. pneumoniae* (CSKP) isolates were defined as susceptible to all three aforementioned carbapenems. Patients with known prior CRKP carriage or infection were excluded to ensure the model predicts new or incident CRKP infection rather than pre-existing colonization. However, carbapenemase (such as KPC, NDM, OXA-48, etc.) typing was not routinely performed in the clinical microbiology laboratory of our hospital during the study period.

### 2.4 Sample size estimation and statistical power

Older rules of thumb demanded ≥10 events per variable, but newer evidence suggests 5–9 can be adequate when penalization methods like LASSO are used (22, 23). With 401 patients and 63 CRKP events (15.7%), the training set had 44 events. After LASSO shrinkage we ended up with roughly 6.3 events per retained variable—comfortably inside the acceptable window.

### 2.5 Statistical analysis

The data missing of all candidate predictors were evaluated. The missing rate of all variables was less than 10%, among which the missing rate of procalcitonin (PCT) was the highest (8.2%), and the missing condition was random missing (MAR). In order to maintain the sample size and avoid bias as much as possible, random forest imputation is used to fill the missing values.

Continuous variables were checked for normality with Shapiro–Wilk; normal data are shown as mean ± SD, others as median (IQR). Categorical variables appear as counts (percentages). Group comparisons used t-tests, Mann–Whitney U, chi-square, or Fisher’s exact tests as appropriate. In the training set, 10-fold cross-validated LASSO selected the penalty *λ* that minimized error and dealt with collinearity (24). The retained variables went into multivariable logistic regression for adjusted odds ratios (95% CI) and into a visual nomogram.

The cohort was randomly split into a training set (70%, *n* = 281) and a validation set (30%, *n* = 120). In the training set, feature selection was performed using 10-fold cross-validated LASSO regression to identify the most parsimonious set of non-redundant predictors (22). The optimal penalty parameter (*λ*) was selected based on the minimum cross-validation error.

For model development, seven machine learning algorithms were trained on the same training set and evaluated on the independent validation set. Hyperparameter tuning was performed using 5-fold cross-validation repeated 3 times for all models to ensure consistency and comparability. The specific tuning strategies were as follows:

#### 2.5.1 Logistic regression (LR)

No hyperparameter tuning; default parameters were used. k-Nearest Neighbors (KNN): Tuned over k (1–30) using 5-fold cross-validation repeated 3 times. Random Forest (RF): Tuned over mtry (2–10) using 5-fold cross-validation repeated 3 times. Decision Tree (DT): Tuned over the complexity parameter (cp) using 5-fold cross-validation repeated 3 times. Neural Network (NN): Tuned over size (3,5,7) and decay (0,0.001,0.01) using 5-fold cross-validation repeated 3 times. XGBoost: Hyperparameters (max_depth, eta, gamma, colsample_bytree, min_child_weight, subsample) were optimized via 5-fold cross-validation with grid search, using early stopping with a patience of 50 rounds based on AUC (25). LightGBM (if available): a similar grid search with 5-fold cross-validation and early stopping (50 rounds) was employed.

All models were ultimately validated on the same independent validation set (*n* = 120) to provide an unbiased comparison of their predictive performance. Discriminatory ability was measured using the area under the receiver operating characteristic curve (AUC). Calibration was evaluated visually with calibration plots and quantitatively using the Brier score. Clinical utility was assessed using decision curve analysis, calculating the net benefit across a range of probability thresholds. The best-performing ML model underwent interpretability analysis using SHAP (SHapley Additive exPlanations) values (26). This provided both global feature importance rankings and local explanations for individual predictions, illustrating how each variable contributed to the model’s output. All statistical analyses were performed using R software (version 4.5.1). A two-sided *p*-value <0.05 was considered statistically significant.

## 3 Results

### 3.1 Cohort characteristics and dataset balance

A total of 401 eligible patients were included, with CRKP identified in 63 (15.7%). The cohort was randomly divided into training (*n* = 281) and validation (*n* = 120) sets. The prevalence of CRKP was nearly identical between the sets (15.7% vs. 15.8%, *p* = 0.965). As detailed in Table 1, the randomization process successfully created balanced sets, with no statistically significant differences observed in baseline demographics, severity scores, laboratory values, procedures, or prior antibiotic exposures (all *p* > 0.05).

**Table 1:** Baseline characteristics and balance assessment between training and validation sets.

Variables	Total (*n* = 401)	Training (*n* = 281)	Validation (*n* = 120)	Statistic	*p*
Age, Mean ± SD	67.421 ± 7.161	67.402 ± 7.135	67.467 ± 7.249	*t* = −0.083	0.934
BMI, M (Q₁, Q₃)	23.000 (22.100, 24.500)	23.000 (22.200, 24.600)	22.850 (22.100, 24.400)	*Z* = −1.021	0.307
Hospital LOS before ICU, M (Q₁, Q₃)	6.000 (3.000, 8.000)	5.000 (3.000, 8.000)	6.000 (3.000, 8.000)	*Z* = −0.732	0.464
Apache II, M (Q₁, Q₃)	16.000 (14.000, 18.000)	16.000 (14.000, 18.000)	16.000 (16.000, 17.000)	*Z* = −0.401	0.689
SOFA, M (Q₁, Q₃)	5.000 (4.000, 6.000)	5.000 (4.000, 6.000)	6.000 (4.000, 6.000)	*Z* = −1.763	0.078
MV days, M (Q₁, Q₃)	3.000 (0.000, 7.000)	2.000 (0.000, 7.000)	3.000 (0.000, 7.000)	*Z* = −1.173	0.241
Albumin, M (Q₁, Q₃)	31.800 (30.300, 33.400)	31.700 (30.300, 33.000)	32.300 (30.775, 33.625)	*Z* = −0.591	0.555
WBC, M (Q₁, Q₃)	13.800 (11.400, 15.700)	13.700 (11.400, 15.600)	14.600 (11.800, 16.400)	*Z* = −1.134	0.257
HB, M (Q₁, Q₃)	107.000 (99.000, 117.000)	106.000 (98.000, 117.000)	109.000 (102.000, 117.250)	*Z* = −0.774	0.439
PLT, M (Q₁, Q₃)	193.000 (166.000, 220.000)	193.000 (169.000, 220.000)	194.500 (165.750, 222.250)	*Z* = −0.431	0.667
Creatinine, M (Q₁, Q₃)	84.000 (72.000, 94.000)	84.000 (72.000, 94.000)	86.000 (74.000, 98.000)	*Z* = −1.325	0.185
PCT, M (Q₁, Q₃)	0.410 (0.320, 0.590)	0.410 (0.320, 0.590)	0.410 (0.320, 0.590)	*Z* = −0.354	0.723
CRP, M (Q₁, Q₃)	18.000 (13.300, 25.400)	18.000 (13.300, 25.400)	18.000 (15.200, 25.400)	*Z* = −0.299	0.765
Gender, *n* (%)				*χ*^2^ = 1.478	0.224
Female	138 (34.414)	102 (36.299)	36 (30.000)		
Male	263 (65.586)	179 (63.701)	84 (70.000)		
Admit source, *n* (%)				*χ*^2^ = 2.848	0.241
Emergency	186 (46.384)	134 (47.687)	52 (43.333)		
Transfer	95 (23.691)	60 (21.352)	35 (29.167)		
Ward	120 (29.925)	87 (30.961)	33 (27.500)		
ICU type, *n* (%)				*χ*^2^ = 3.236	0.198
Medical	150 (37.406)	106 (37.722)	44 (36.667)		
Surgical	186 (46.384)	124 (44.128)	62 (51.667)		
Trauma	65 (16.209)	51 (18.149)	14 (11.667)		
Hypertension, *n* (%)				*χ*^2^ = 0.472	0.492
0	221 (55.112)	158 (56.228)	63 (52.500)		
1	180 (44.888)	123 (43.772)	57 (47.500)		
Diabetes, *n* (%)				*χ*^2^ = 0.468	0.494
0	316 (78.803)	224 (79.715)	92 (76.667)		
1	85 (21.197)	57 (20.285)	28 (23.333)		
CHD, *n* (%)				*χ*^2^ = 0.005	0.944
0	330 (82.294)	231 (82.206)	99 (82.500)		
1	71 (17.706)	50 (17.794)	21 (17.500)		
CKD, *n* (%)				*χ*^2^ = 1.258	0.262
0	373 (93.017)	264 (93.950)	109 (90.833)		
1	28 (6.983)	17 (6.050)	11 (9.167)		
Malignancy, *n* (%)				*χ*^2^ = 0.455	0.500
0	382 (95.262)	269 (95.730)	113 (94.167)		
1	19 (4.738)	12 (4.270)	7 (5.833)		
COPD, *n* (%)				*χ*^2^ = 0.069	0.793
0	355 (88.529)	248 (88.256)	107 (89.167)		
1	46 (11.471)	33 (11.744)	13 (10.833)		
Immunosuppression, *n* (%)				*χ*^2^ = 0.034	0.854
0	387 (96.509)	272 (96.797)	115 (95.833)		
1	14 (3.491)	9 (3.203)	5 (4.167)		
Liver disease, *n* (%)				*χ*^2^ = 0.020	0.887
0	392 (97.756)	274 (97.509)	118 (98.333)		
1	9 (2.244)	7 (2.491)	2 (1.667)		
Stroke history, *n* (%)				*χ*^2^ = 0.323	0.570
0	386 (96.259)	269 (95.730)	117 (97.500)		
1	15 (3.741)	12 (4.270)	3 (2.500)		
Mech ventilation, *n* (%)				*χ*^2^ = 0.038	0.845
0	160 (39.900)	113 (40.214)	47 (39.167)		
1	241 (60.100)	168 (59.786)	73 (60.833)		
CVC, *n* (%)				–	1.000
0	1 (0.249)	1 (0.356)	0 (0.00)		
1	400 (99.751)	280 (99.644)	120 (100.000)		
NG tube, *n* (%)				*χ*^2^ = 1.646	0.200
0	163 (40.648)	120 (42.705)	43 (35.833)		
1	238 (59.352)	161 (57.295)	77 (64.167)		
Drainage tube, *n* (%)				*χ*^2^ = 0.711	0.399
0	368 (91.771)	260 (92.527)	108 (90.000)		
1	33 (8.229)	21 (7.473)	12 (10.000)		
Prior carbapenems, *n* (%)				*χ*^2^ = 0.180	0.672
0	371 (92.519)	261 (92.883)	110 (91.667)		
1	30 (7.481)	20 (7.117)	10 (8.333)		
Prior cephalosporins, *n* (%)				*χ*^2^ = 1.257	0.262
0	244 (60.848)	176 (62.633)	68 (56.667)		
1	157 (39.152)	105 (37.367)	52 (43.333)		
Prior fluoroquinolones, *n* (%)				*χ*^2^ = 0.010	0.919
0	333 (83.042)	233 (82.918)	100 (83.333)		
1	68 (16.958)	48 (17.082)	20 (16.667)		
Prior penicillins, n (%)				*χ*^2^ = 0.440	0.507
0	280 (69.825)	199 (70.819)	81 (67.500)		
1	121 (30.175)	82 (29.181)	39 (32.500)		
Prior aminoglycosides, *n* (%)				*χ*^2^ = 0.000	1.000
0	396 (98.753)	277 (98.577)	119 (99.167)		
1	5 (1.247)	4 (1.423)	1 (0.833)		
History MDR infection, *n* (%)				*χ*^2^ = 0.000	1.000
0	390 (97.257)	273 (97.153)	117 (97.500)		
1	11 (2.743)	8 (2.847)	3 (2.500)		
Specimen type, *n* (%)				–	0.886
Blood	91 (22.693)	64 (22.776)	27 (22.500)		
Drainage	6 (1.496)	4 (1.423)	2 (1.667)		
Sputum	257 (64.090)	182 (64.769)	75 (62.500)		
Urine	47 (11.721)	31 (11.032)	16 (13.333)		
CRKP, *n* (%)				*χ*^2^ = 0.002	0.965
0	338 (84.289)	237 (84.342)	101 (84.167)		
1	63 (15.711)	44 (15.658)	19 (15.833)		

*t*, *t*-test; *Z*, Mann–Whitney test; *χ*^2^, Chi-square test; −, Fisher exact.

SD, standard deviation; M, Median; Q₁, 1st Quartile; Q₃, 3rd Quartile; 0, No; 1, Yes.

### 3.2 Univariate analysis in the training set

In the training set (*n* = 281), patients with CRKP (*n* = 44) were significantly older and had higher disease severity scores (APACHE II and SOFA) compared to those with CSKP (*n* = 237) (Table 2). Markers of systemic inflammation were also elevated in the CRKP group, with procalcitonin (PCT) showing a particularly pronounced difference (median 0.545 vs. 0.410 ng/mL, *p* < 0.05). Key exposure histories were strongly associated with CRKP: prior carbapenem use within 90 days (15.9% vs. 5.5%, *p* = 0.032) and a history of MDR infection (9.1% vs. 1.7%, *p* = 0.027). Furthermore, CRKP was significantly more likely to be isolated from blood cultures (59.1% vs. 16.0%, *p* < 0.001).

**Table 2:** Univariate analysis of risk factors for CRKP in training set.

Variables	Total (*n* = 281)	CSKP (*n* = 237)	CRKP (*n* = 44)	Statistic	*p*
Age, Mean ± SD	67.402 ± 7.135	66.899 ± 7.189	70.114 ± 6.240	*t* = −2.777	0.006
BMI, M (Q₁, Q₃)	23.000 (22.200, 24.600)	23.000 (22.200, 24.500)	23.050 (22.375, 24.625)	*Z* = −0.435	0.663
Hospital LOS before ICU, M (Q₁, Q₃)	5.000 (3.000, 8.000)	5.000 (3.000, 8.000)	5.000 (3.000, 7.000)	*Z* = −0.144	0.886
APACHE II, M (Q₁, Q₃)	16.000 (14.000, 18.000)	16.000 (14.000, 18.000)	18.000 (16.000, 26.000)	*Z* = −3.933	<0.001
SOFA, M (Q₁, Q₃)	5.000 (4.000, 6.000)	5.000 (4.000, 6.000)	6.000 (4.000, 6.000)	*Z* = −2.058	0.040
MV days, M (Q₁, Q₃)	2.000 (0.000, 7.000)	2.000 (0.000, 7.000)	3.000 (1.000, 7.000)	*Z* = −0.574	0.566
Albumin, M (Q₁, Q₃)	31.700 (30.300, 33.000)	31.800 (30.300, 33.400)	31.550 (29.975, 32.625)	*Z* = −1.444	0.149
WBC, M (Q₁, Q₃)	13.700 (11.400, 15.600)	13.300 (11.300, 15.600)	14.700 (13.200, 15.125)	*Z* = −2.442	0.015
HB, M (Q₁, Q₃)	106.000 (98.000, 117.000)	107.000 (100.000, 118.000)	105.000 (95.000, 113.000)	*Z* = −1.990	0.047
PLT, M (Q₁, Q₃)	193.000 (169.000, 220.000)	193.000 (169.000, 220.000)	196.000 (161.750, 208.000)	*Z* = −0.801	0.423
Creatinine, M (Q₁, Q₃)	84.000 (72.000, 94.000)	84.000 (72.000, 94.000)	87.000 (81.000, 96.750)	*Z* = −1.909	0.056
PCT, M (Q₁, Q₃)	0.410 (0.320, 0.590)	0.410 (0.320, 0.570)	0.545 (0.315, 1.100)	*Z* = −1.984	0.047
CRP, M (Q₁, Q₃)	18.000 (13.300, 25.400)	18.000 (13.300, 25.400)	19.300 (12.900, 25.825)	*Z* = −0.616	0.538
Gender, *n* (%)				*χ*^2^ = 0.123	0.726
Female	102 (36.299)	85 (35.865)	17 (38.636)		
Male	179 (63.701)	152 (64.135)	27 (61.364)		
Admit source, *n* (%)				*χ*^2^ = 0.948	0.623
Emergency	134 (47.687)	112 (47.257)	22 (50.000)		
Transfer	60 (21.352)	53 (22.363)	7 (15.909)		
Ward	87 (30.961)	72 (30.380)	15 (34.091)		
ICU type, *n* (%)				*χ*^2^ = 2.997	0.223
Medical	106 (37.722)	92 (38.819)	14 (31.818)		
Surgical	124 (44.128)	106 (44.726)	18 (40.909)		
Trauma	51 (18.149)	39 (16.456)	12 (27.273)		
Hypertension, *n* (%)				*χ*^2^ = 0.060	0.807
0	158 (56.228)	134 (56.540)	24 (54.545)		
1	123 (43.772)	103 (43.460)	20 (45.455)		
Diabetes, *n* (%)				*χ*^2^ = 2.568	0.109
0	224 (79.715)	185 (78.059)	39 (88.636)		
1	57 (20.285)	52 (21.941)	5 (11.364)		
CHD, *n* (%)				*χ*^2^ = 0.005	0.942
0	231 (82.206)	195 (82.278)	36 (81.818)		
1	50 (17.794)	42 (17.722)	8 (18.182)		
CKD, *n* (%)				*χ*^2^ = 0.000	1.000
0	264 (93.950)	223 (94.093)	41 (93.182)		
1	17 (6.050)	14 (5.907)	3 (6.818)		
Malignancy, *n* (%)				*χ*^2^ = 0.254	0.614
0	269 (95.730)	228 (96.203)	41 (93.182)		
1	12 (4.270)	9 (3.797)	3 (6.818)		
COPD, *n* (%)				*χ*^2^ = 0.180	0.671
0	248 (88.256)	210 (88.608)	38 (86.364)		
1	33 (11.744)	27 (11.392)	6 (13.636)		
Immunosuppression, *n* (%)				*χ*^2^ = 3.799	0.051
0	272 (96.797)	232 (97.890)	40 (90.909)		
1	9 (3.203)	5 (2.110)	4 (9.091)		
Liver disease, *n* (%)				*χ*^2^ = 0.000	1.000
0	274 (97.509)	231 (97.468)	43 (97.727)		
1	7 (2.491)	6 (2.532)	1 (2.273)		
Stroke history, *n* (%)				*χ*^2^ = 1.732	0.188
0	269 (95.730)	229 (96.624)	40 (90.909)		
1	12 (4.270)	8 (3.376)	4 (9.091)		
Mech ventilation, *n* (%)				*χ*^2^ = 1.529	0.216
0	113 (40.214)	99 (41.772)	14 (31.818)		
1	168 (59.786)	138 (58.228)	30 (68.182)		
CVC, *n* (%)				–	1.000
0	1 (0.356)	1 (0.422)	0 (0.00)		
1	280 (99.644)	236 (99.578)	44 (100.000)		
NG tube, *n* (%)				*χ*^2^ = 0.353	0.552
0	120 (42.705)	103 (43.460)	17 (38.636)		
1	161 (57.295)	134 (56.540)	27 (61.364)		
Drainage tube, *n* (%)				*χ*^2^ = 0.000	1.000
0	260 (92.527)	219 (92.405)	41 (93.182)		
1	21 (7.473)	18 (7.595)	3 (6.818)		
Prior carbapenems, *n* (%)				*χ*^2^ = 4.625	0.032
0	261 (92.883)	224 (94.515)	37 (84.091)		
1	20 (7.117)	13 (5.485)	7 (15.909)		
Prior cephalosporins, *n* (%)				*χ*^2^ = 0.754	0.385
0	176 (62.633)	151 (63.713)	25 (56.818)		
1	105 (37.367)	86 (36.287)	19 (43.182)		
Prior fluoroquinolones, *n* (%)				*χ*^2^ = 0.045	0.833
0	233 (82.918)	197 (83.122)	36 (81.818)		
1	48 (17.082)	40 (16.878)	8 (18.182)		
Prior penicillins, *n* (%)				*χ*^2^ = 0.003	0.954
0	199 (70.819)	168 (70.886)	31 (70.455)		
1	82 (29.181)	69 (29.114)	13 (29.545)		
Prior aminoglycosides, *n* (%)				–	0.496
0	277 (98.577)	234 (98.734)	43 (97.727)		
1	4 (1.423)	3 (1.266)	1 (2.273)		
History MDR infection, *n* (%)				*χ*^2^ = 4.920	0.027
0	273 (97.153)	233 (98.312)	40 (90.909)		
1	8 (2.847)	4 (1.688)	4 (9.091)		
Specimen type, *n* (%)				–	<0.001
Blood	64 (22.776)	38 (16.034)	26 (59.091)		
Drainage	4 (1.423)	3 (1.266)	1 (2.273)		
Sputum	182 (64.769)	168 (70.886)	14 (31.818)		
Urine	31 (11.032)	28 (11.814)	3 (6.818)		

*t*, *t*-test; *Z*, Mann–Whitney test; *χ*^2^, Chi-square test; −, Fisher exact.

SD, standard deviation; M, Median; Q₁, 1st Quartile; Q₃, 3rd Quartile.

### 3.3 Feature selection via LASSO regression

**Figure 2:**
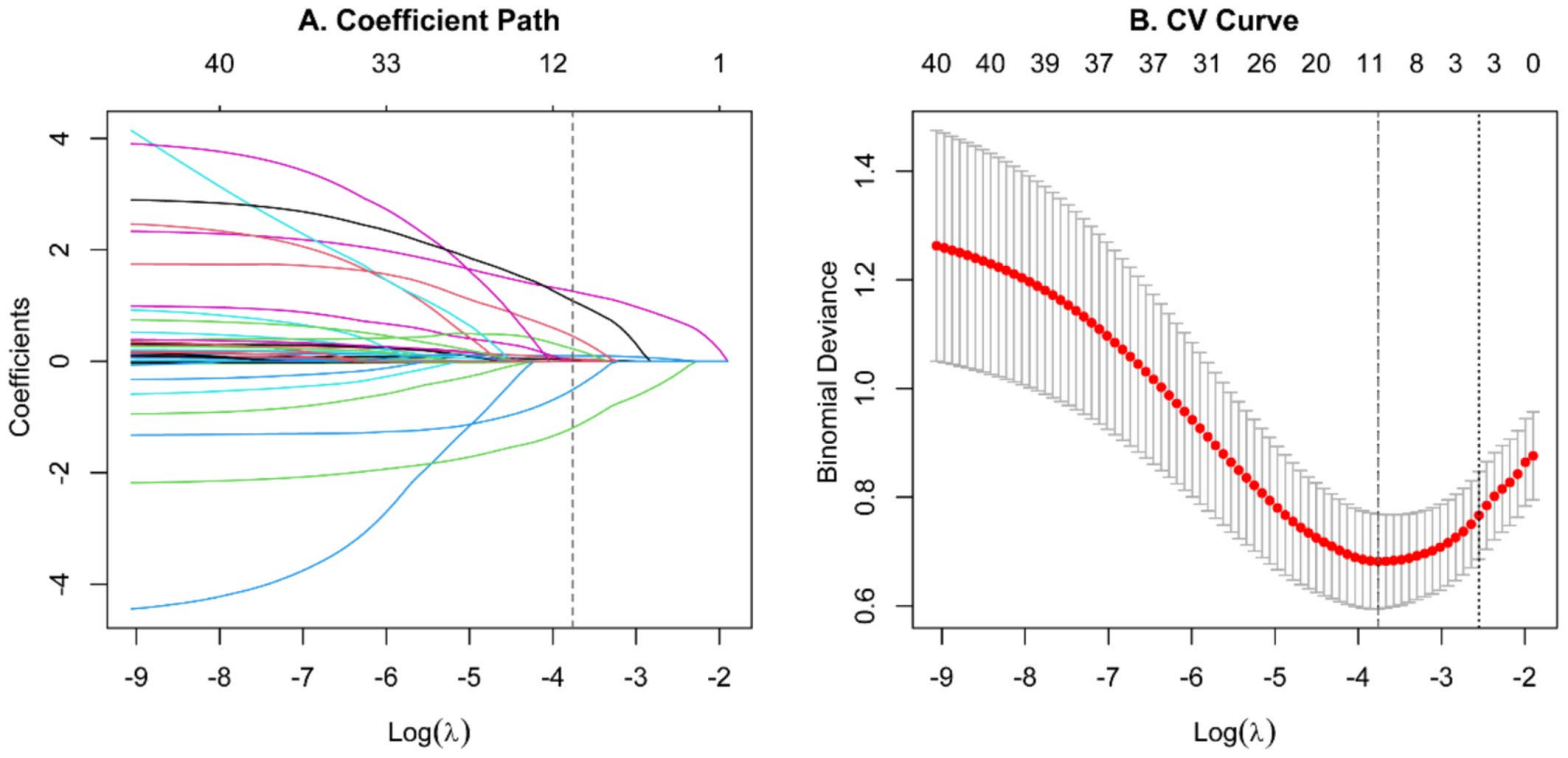
LASSO feature selection. **(A)** Coefficient paths as log(*λ*) increases. **(B)** Cross-validation error curve. Dashed lines indicate log(λ_min) = −3.772, corresponding to λ_min = 0.023. The chosen model retained nine features.

LASSO regression applied to the training set effectively reduced the initial set of candidate variables. The cross-validation curve indicated the optimal lambda (*λ*.min) value, at which nine variables retained non-zero coefficients (Figure 2). The selected features, in descending order of absolute coefficient value, were: PCT, specimen type (sputum), history of MDR infection, specimen type (urine), prior carbapenem exposure, history of stroke, APACHE II score, WBC, age, and hemoglobin (Table 3).

**Table 3:** Features selected by LASSO regression and their coefficients.

Feature	Coef	Abs_Coef
PCT	1.257	1.257
Specimen_typeSputum	−1.194	1.194
History_MDR_infection1	1.083	1.083
Specimen_typeUrine	−0.509	0.509
Prior_carbapenems1	0.451	0.451
Stroke_history1	0.222	0.222
APACHE_II	0.099	0.099
WBC	0.047	0.047
Age	0.031	0.031
HB	−0.007	0.007

### 3.4 Multivariable logistic regression

When the nine LASSO-selected variables were included in a multivariable logistic regression model, history of MDR infection (OR = 11.65, 95% CI: 1.18–115.15), elevated PCT (OR = 7.18, 95% CI: 2.08–24.77), and prior carbapenem exposure (OR = 4.68, 95% CI: 1.12–19.65) emerged as the strongest independent risk factors (Table 4). Increasing age and APACHE II score were also significant predictors. Compared to blood cultures, isolates from sputum (OR = 0.13) and urine (OR = 0.19) were associated with a substantially lower risk of CRKP.

**Table 4:** Multivariate logistic regression analysis for CRKP prediction.

Variables	*β*	S.E	*Z*	*p*	OR (95%CI)
Specimen type					
Blood					1.000 (Reference)
Drainage	0.522	1.316	0.397	0.691	1.686 (0.128 ~ 22.241)
Sputum	−2.070	0.487	−4.246	<0.001	0.126 (0.049 ~ 0.328)
Urine	−1.688	0.811	−2.081	0.037	0.185 (0.038 ~ 0.907)
APACHE II	0.118	0.050	2.335	0.020	1.125 (1.019 ~ 1.241)
SOFA	0.108	0.164	0.658	0.510	1.114 (0.808 ~ 1.536)
WBC	0.211	0.093	2.258	0.024	1.234 (1.028 ~ 1.482)
Age	0.079	0.033	2.369	0.018	1.082 (1.014 ~ 1.156)
HB	−0.026	0.017	−1.527	0.127	0.975 (0.943 ~ 1.007)
PCT	1.971	0.632	3.119	0.002	7.178 (2.080 ~ 24.769)
Prior carbapenems					
0					1.000 (Reference)
1	1.544	0.732	2.109	0.035	4.681 (1.116 ~ 19.645)
History MDR infection					
0					1.000 (Reference)
1	2.455	1.169	2.100	0.036	11.648 (1.178 ~ 115.151)

OR, Odds Ratio; CI, Confidence Interval; 0, No; 1, Yes.

### 3.5 Nomogram development

**Figure 3:**
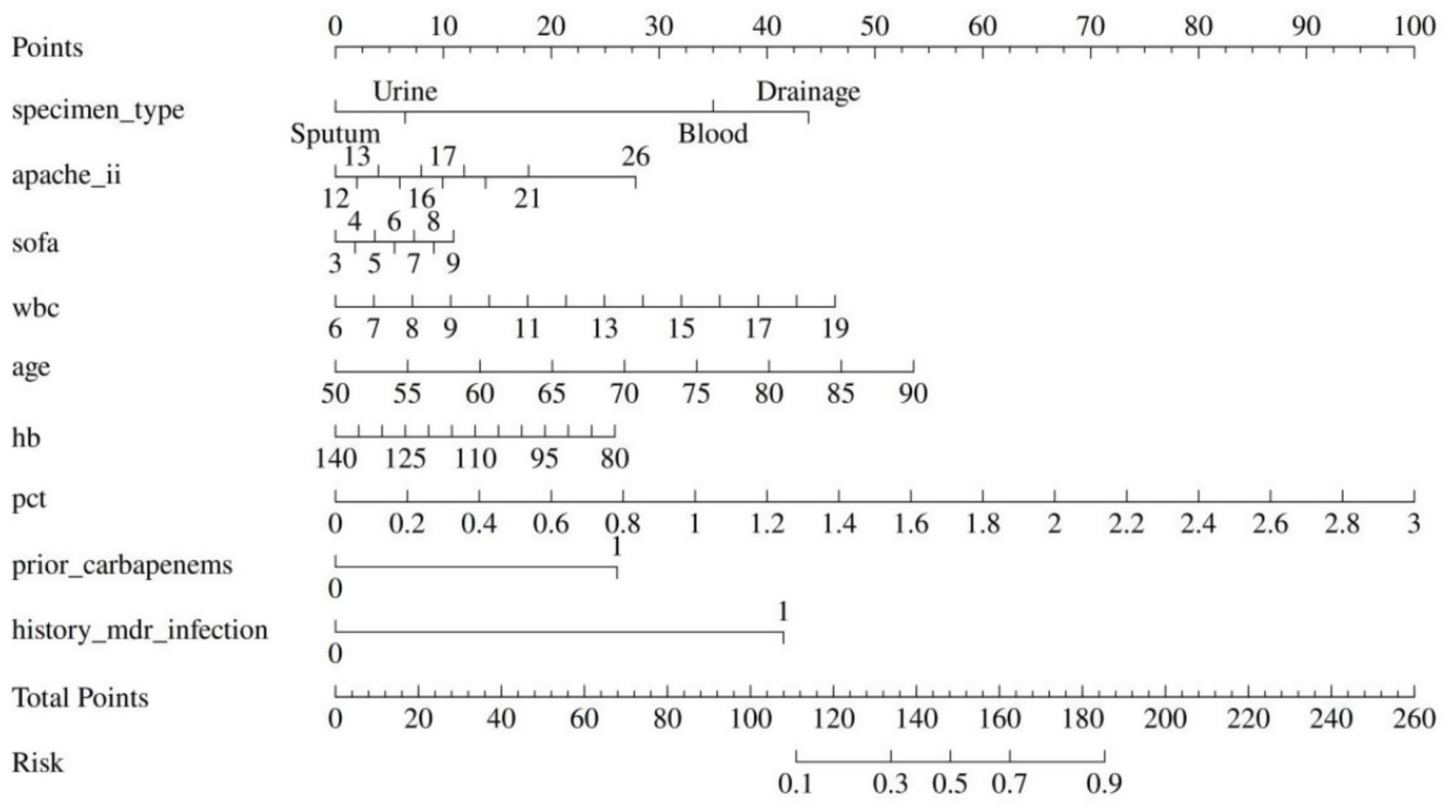
Nomogram based on multivariable logistic regression.

The logistic model translated directly into an easy-to-use nomogram that includes APACHE II, PCT, prior carbapenem and MDR history, and specimen type (Figure 3). Adding the points gives the predicted probability.

### 3.6 Machine learning model performance comparison

**Figure 4:**
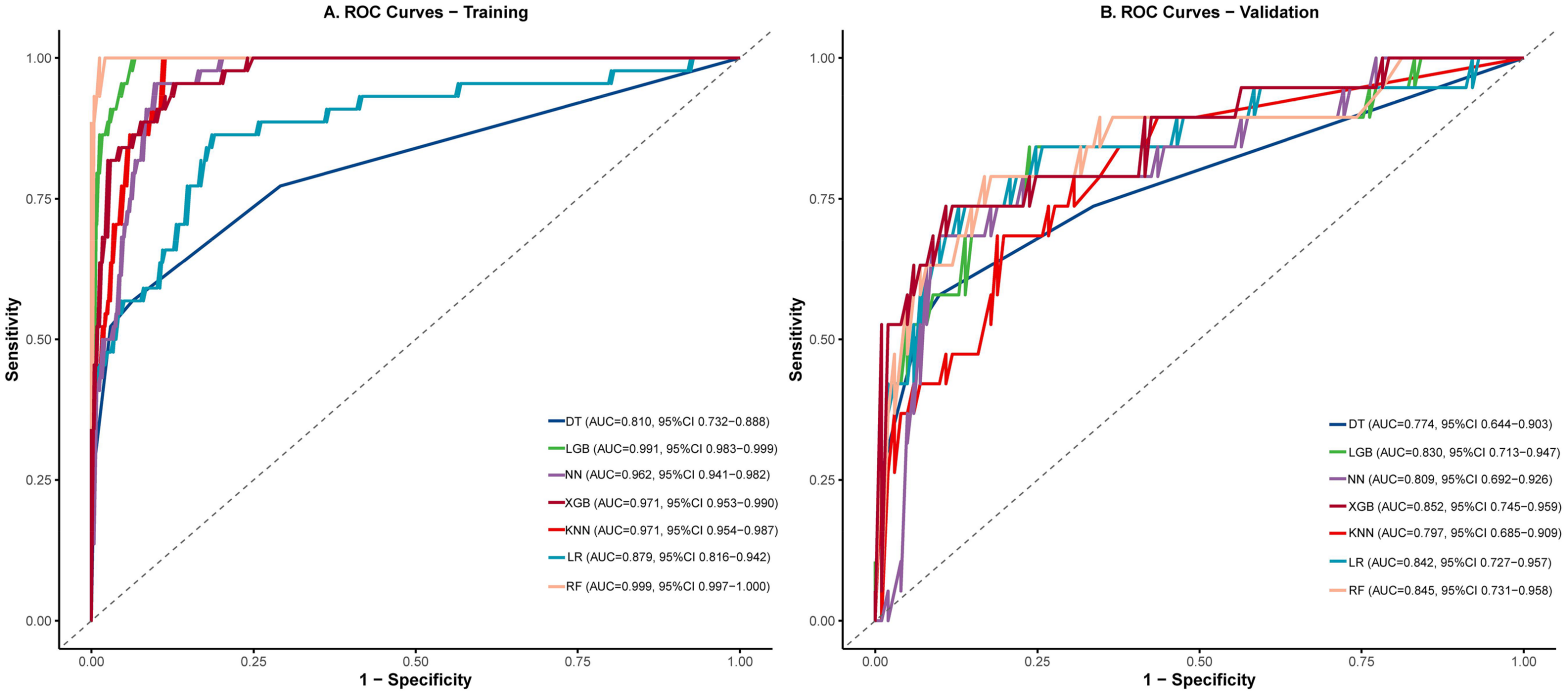
ROC curves for all models: **(A)** Training set and **(B)** validation set.

To further improve the prediction accuracy, we evaluated and compared the performance of seven machine learning algorithms, including Logistic Regression (LR), XGBoost, Random Forest (RF), and LightGBM. All models were trained on the same training set and subsequently tested on an independent validation set. The results indicate that the XGBoost model demonstrated the most consistent discriminative ability, achieving an AUC of 0.971 on the training set and 0.852 on the validation set, as shown in Figure 4.

On the validation set, RF and LGB exhibited the largest decreases in AUC from training to validation (dropping by 0.154 and 0.161, respectively), suggesting potential overfitting during training. Although RF’s metrics were nearly perfect on the training set, its PPV (0.469) and F1-score (0.588) declined substantially on the validation set. In contrast, XGBoost showed the most robust performance on the validation set, with a relatively smaller AUC decrease (0.119), and achieved the highest validation AUC, good specificity (0.891), and the highest F1-score (0.636). LR also demonstrated better generalization, with the smallest AUC drop (only 0.037), rising from the 6th rank on the training set to the 3rd on the validation set. While the Neural Network (NN) had a mid-range AUC ranking, its optimal threshold on the validation set (0.669) was much higher than that on the training set (0.195). At this threshold, it showed higher specificity (0.911) and PPV (0.591), but lower sensitivity (0.684). Both KNN and DT achieved AUCs below 0.800 on the validation set, indicating relatively poorer overall performance (Table 5).

**Table 5:** Performance metrics comparison of seven machine learning models.

Model	Set	AUC	AUC 95%CI	Sensitivity	Specificity	Accuracy	PPV	NPV	F1
XGB	Validation	0.852	0.745–0.959	0.737	0.891	0.867	0.56	0.947	0.636
RF	Validation	0.845	0.731–0.958	0.789	0.832	0.825	0.469	0.955	0.588
LR	Validation	0.842	0.727–0.957	0.737	0.871	0.85	0.519	0.946	0.609
LGB	Validation	0.83	0.713–0.947	0.842	0.762	0.775	0.4	0.963	0.542
NN	Validation	0.809	0.692–0.926	0.684	0.911	0.875	0.591	0.939	0.634
KNN	Validation	0.797	0.685–0.909	0.684	0.812	0.792	0.406	0.932	0.51
DT	Validation	0.774	0.644–0.903	0.579	0.901	0.85	0.524	0.919	0.55

### 3.7 Model calibration and clinical benefit

**Figure 5:**
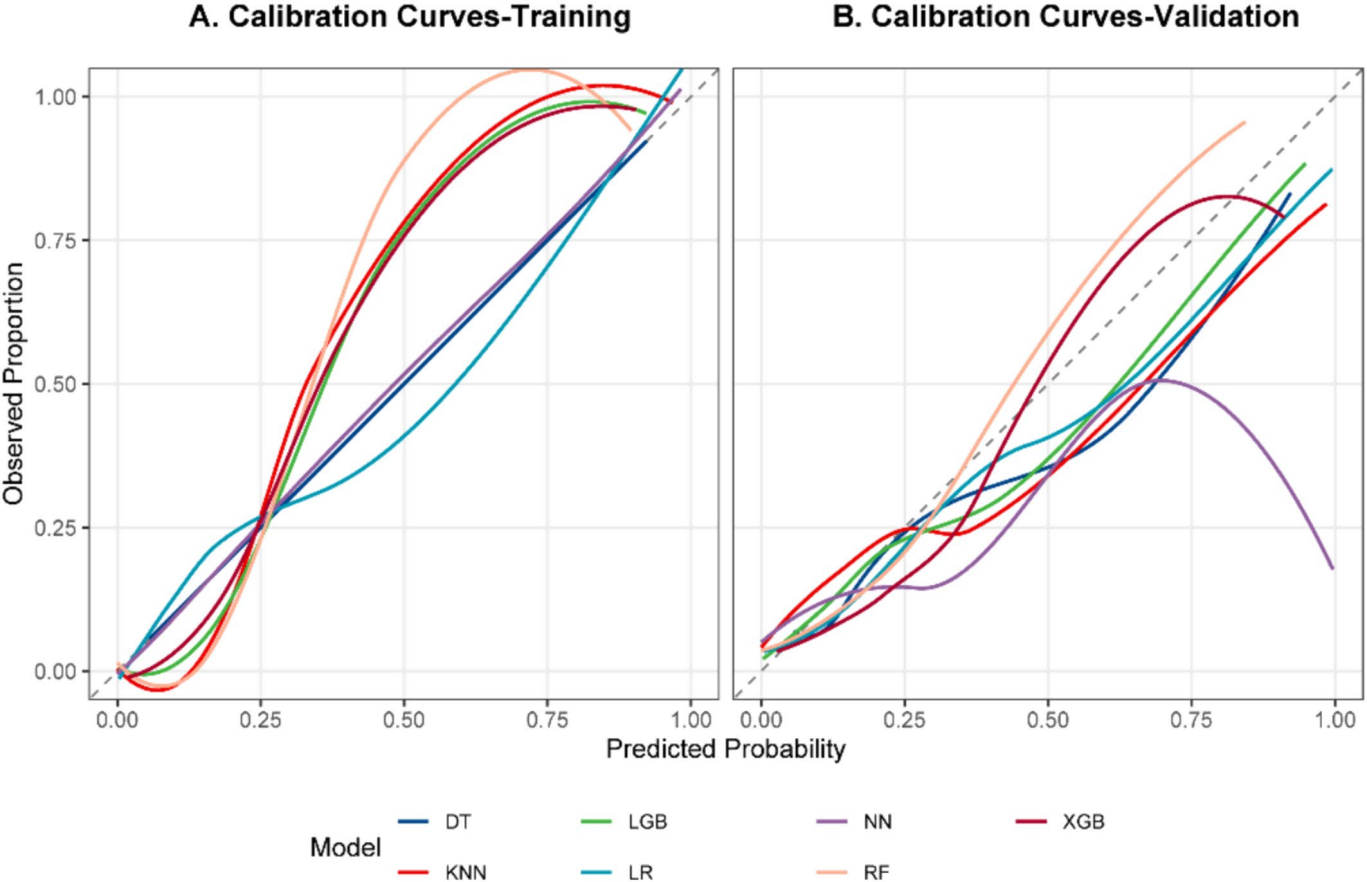
Calibration curves. **(A)** Training. **(B)** Validation.

**Figure 6:**
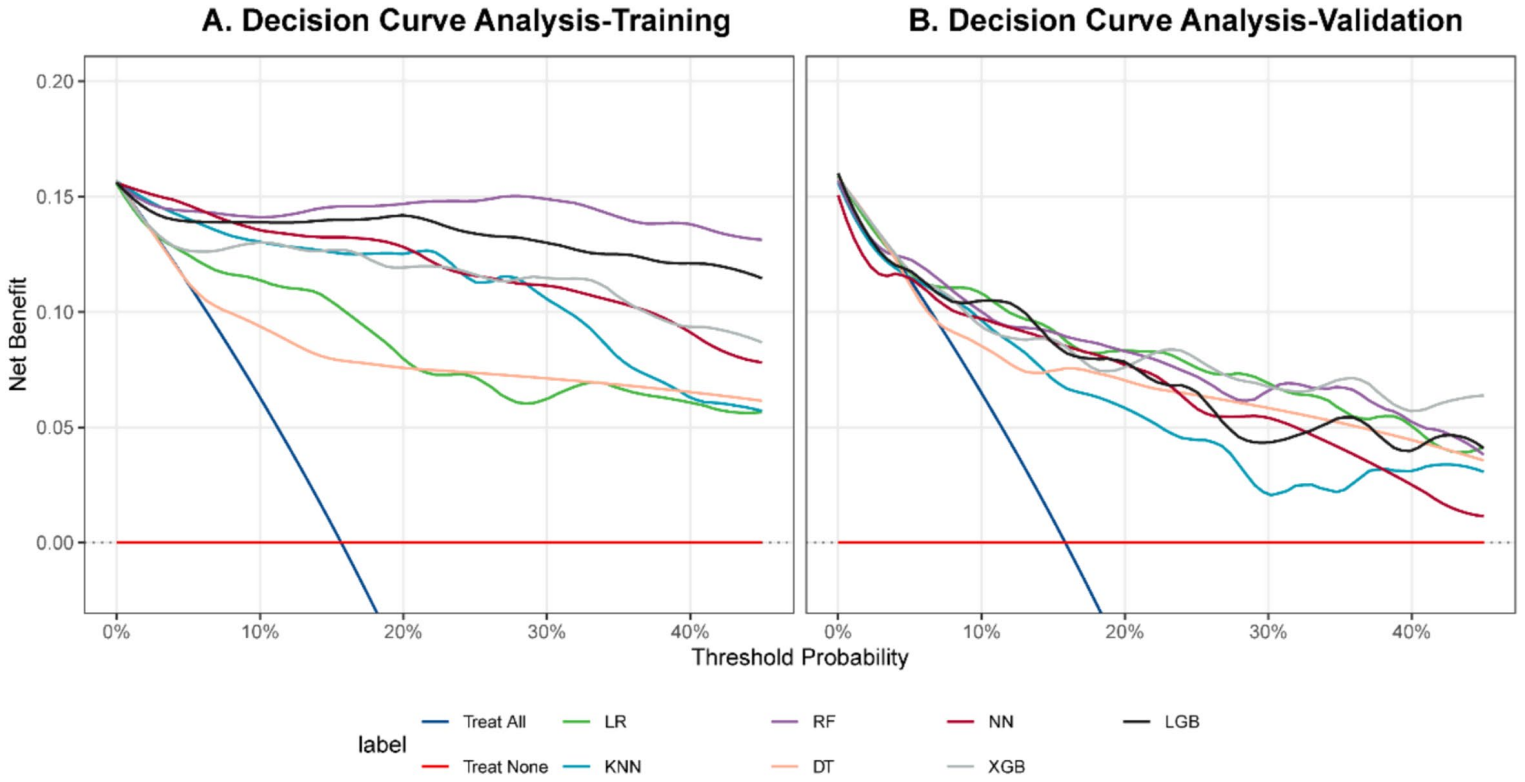
Decision curve analysis. **(A)** Training. **(B)** Validation.

In validation, XGBoost tracked the ideal calibration line most closely (Figure 5), posting the lowest Brier score (0.088) and a slope close to 1 (1.222; Table 6), and its mean absolute error (MAE) was calculated as 0.0629. Decision curve analysis showed net benefit over treat-all or treat-none approaches across plausible thresholds (Figure 6).

**Table 6:** Calibration metrics across models.

Set	Model	Brier	Cal_Intercept	Cal_Slope
Validation	DT	0.101	−0.527	0.759
Validation	KNN	0.108	−0.774	0.218
Validation	LGB	0.095	−0.382	0.781
Validation	LR	0.09	−0.534	0.707
Validation	NN	0.121	−0.786	0.277
Validation	RF	0.092	0.056	0.882
Validation	XGB	0.088	0.114	1.222

### 3.8 SHAP-based interpretability

**Figure 7:**
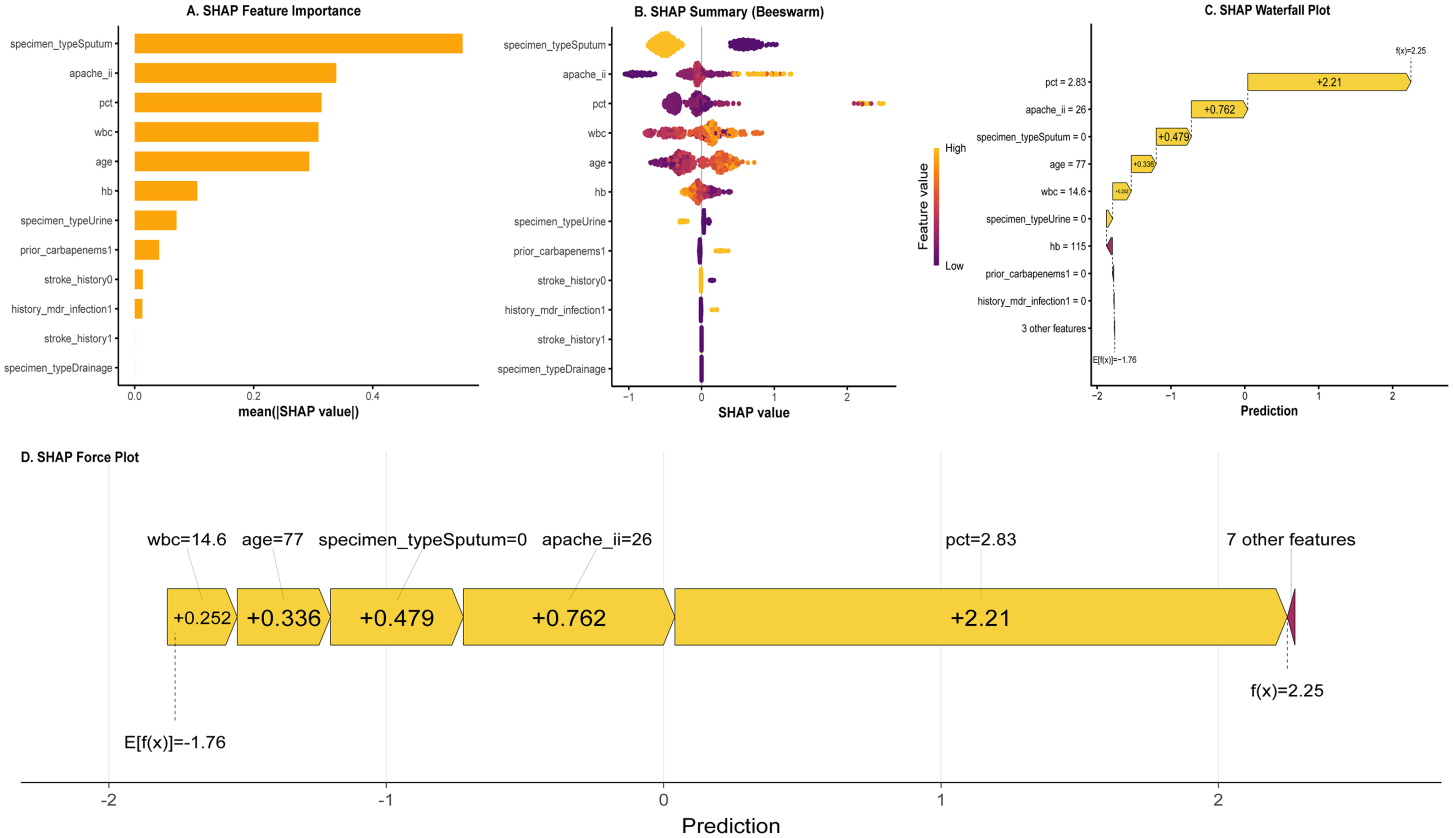
SHAP explanations for the XGBoost model. **(A)** Global feature importance. **(B)** Summary beeswarm plot. **(C)** Waterfall plot for one patient. **(D)** Force plot for one patient.

On the validation set, XGBoost demonstrated the best discriminative ability (highest AUC), the most reliable probability calibration (the lowest Brier score), and the most robust clinical utility. This indicates that its predictive outcomes not only classify patients correctly but also generate risk probability scores with substantial clinical reference value. Despite its superior performance, the complex tree structure of XGBoost is often difficult for clinicians to interpret intuitively. To address this, we employed SHAP analysis to decipher the model, with the results presented in Figure 7.

As shown in Figure 7A, the feature importance ranking reveals that Specimen Type (sputum), APACHE II score, Procalcitonin (PCT), White Blood Cell count (WBC), and Age are the most critical features for the model’s predictions. The SHAP summary plot (Beeswarm Plot) in Figure 7B further elucidates the relationship between feature values and predicted risk. The results indicate that higher APACHE II scores, PCT levels, WBC counts, and advanced age are significantly associated with positive SHAP values, confirming these factors as risk factors for the target outcome.

Individual case visualizations using waterfall plots (Figure 7C) and force plots (Figure 7D) illustrate how the model arrives at a prediction for a single patient. Taking this representative patient as an example, the base value (E[f(x)]) was −1.76, and the final model output (f(x)) increased to 2.25. Here, PCT = 2.83 (contributing +2.21) and APACHE II = 26 (contributing +0.762) were the primary drivers elevating the predicted probability. This individualized interpretation verifies a high degree of consistency between the model’s predictions and established clinical logic.

## 4 Discussion

In this cohort of 401 ICU patients with confirmed *K. pneumoniae*, CRKP prevalence came in at 15.7%—broadly in line with recent regional figures, though inevitably influenced by local ecology and specimen mix (27, 28). Using only routine data, the LASSO–XGBoost combination achieved a respectable validation AUC of 0.852 for risk assessment before susceptibility results were available.

### 4.1 Association of core predictors with CRKP risk

The predictors identified by our model are firmly grounded in established mechanisms of antimicrobial resistance and host-pathogen interaction. Prior carbapenem exposure and selective pressure. In our study, prior exposure to carbapenems was associated with a markedly higher risk of CRKP (OR 4.681), consistent with the findings reported by Tumbarello et al. (29) and Wu et al. (30). Evidence from previous meta-analyses also supports the view that exposure to carbapenems or other broad-spectrum antibiotics constitutes a key risk factor for CRKP infection, plausibly because carbapenems represent a class of agents exerting particularly strong selective pressure. By suppressing susceptible commensal flora and perturbing the intestinal microecological barrier, such exposure can create ecological space in which strains carrying resistance determinants gain a competitive advantage for colonization; under conditions of critical illness, these colonizing organisms may subsequently transition into endogenous infection. Taken together, this pattern accords well with the “antibiotic selective pressure” hypothesis, in which antimicrobial exposure promotes the establishment and expansion of resistant strains within the host (31).

Prior MDR infection history and colonization-to-infection transition. A history of MDR infection emerged as the highest-weight predictor in our model (OR 11.648). Clinically, such a history often signals that the patient has already experienced resistant-organism colonization, or has undergone repeated exposure to environments with intense resistance pressure. In recent years, the linkage between colonization and subsequent infection has been emphasized repeatedly: CRKP can persistently colonize sites such as the gastrointestinal tract, and this carriage state appears closely connected to later extraintestinal infection (32). Kontopoulou et al. (33) reported that intestinal colonization with CRKP may persist for months and that colonization acts as an independent risk factor for subsequent bloodstream infection. In parallel, Tofarides et al. (34) observed a strong concordance between a patient’s prior resistant-infection history and the resistant phenotype observed in the current infection episode. These observations collectively suggest that patients with a documented “resistance record” may harbor a long-standing internal reservoir of resistant organisms, which can readily drive recurrence once host defenses are compromised.

Specificity of Procalcitonin (PCT): CRKP-associated infections frequently occur in high-risk hosts, involve greater exposure, and are more prone to delays or failures in treatment, thereby presenting with more pronounced systemic inflammatory responses and higher bacterial burden signals. Our model incorporated PCT (OR 7.178), aligning with findings by Gao et al. (35) that markedly elevated PCT does not merely indicate infection in general, but may be more suggestive—albeit not definitive—of drug-resistant Gram-negative infection. One plausible explanation is that resistant organisms may sustain inflammatory stimulation through greater or more persistent release of endotoxin, particularly lipopolysaccharide (LPS), thereby amplifying the downstream inflammatory cascade; moreover, CRKP infections are often accompanied by higher bacterial loads and a higher likelihood of treatment delay or failure, which may further drive PCT elevation (36).

Beyond prior carbapenem exposure, history of multidrug-resistant infection, and PCT, factors such as APACHE II score, age, white blood cell count, and specimen type also exert independent or synergistic influences on CRKP risk. An elevated APACHE II score reflects critical illness and exposure to broad-spectrum therapies, factors that promote selective pressure and are linked to mortality in bloodstream infections (10, 11). Advanced age may predispose patients to resistant pathogen colonization and subsequent progression to invasive infection through mechanisms like immunosenescence and gut microbiota dysbiosis (37). While a non-specific inflammatory marker, an elevated white blood cell count in the context of severe infection can signal a persistent bacterial burden, and together with PCT, indicates a heightened inflammatory state (35). Compared to blood specimens, sputum and urine sources demonstrated a “protective effect,” suggesting that CRKP is more frequently concentrated in highly invasive scenarios such as bloodstream infections (38).

### 4.2 Comparison with existing models

When set against five contemporary prediction tools (Table 7), our model stands out for relying solely on routinely available variables, avoiding esoteric markers such as CD4/CD8 ratios (39). The strict ICU focus and inclusion of strong predictors like MDR history and PCT gave us a higher validation AUC than models built on mixed ward/ICU populations [0.852 vs. 0.788 (16)]. Comparing multiple algorithms also adds confidence beyond what specialty-restricted models could offer (13, 14). At the same time, Shan et al. (17) also proved that XGBoost is superior to other algorithms in predicting morbidity. Although their research focuses on sepsis, the similarities in methods strengthen the robustness of our methods. However, this study lacks external validation, needs to consider the potential bias in cross-study comparisons, and needs to be interpreted cautiously in numerical comparisons.

**Table 7:** Comparison with representative prior CRKP prediction models.

Dimension	Chu et al. (2024) (13)	Lu et al. (2024) (14)	Li Y et al. (2019) (39)	Xu et al. (2025) (16)	This study
Study population	Pediatric patients (0–14 years)	Neuro-ICU	General ICU	Hospitalized patients (including general wards)	Adult general ICU
Sample size	185	544	507	1,113	401
Modeling method	Logistic Regression	Logistic Regression	Logistic Regression	LASSO + Logistic Regression	LASSO + 7 Machine Learning Algorithms
Model performance (AUC)	0.872	0.907	0.854	0.788	0.852
Key limitations	Pediatric cohort; findings may not generalize to adults	Specific to a specialized ICU population	Reliance on non-routine biomarkers (e.g., CD4/CD8)	Heterogeneous cohort may limit specificity	Single-center retrospective design with limited sample size; lacks external validation.

### 4.3 Clinical translation and stewardship implications

The PPV of 0.567 in the validation set of this model showed that 56.7% of the patients with high risk of the model did have CRKP. Although this indicates a false positive rate of about 43%, there are several considerations that can place this finding in the expected clinical use case. First, in the absence of a model, clinicians face a treatment dilemma, with a baseline CRKP prevalence of 15.7% in our intensive care unit. The discovery probability of the model after summarizing the early clinical information reaches 56.7%, which is actually a large information gain and can better guide clinical decision-making. Second, decision curve analysis (Figure 6) shows that the model provides a net clinical benefit over a range of risk thresholds compared to a ‘treat all ‘or ‘treat none ‘strategy, indicating that there is a threshold within which the benefit of early appropriate treatment is greater than the harm of overtreatment. Third, the model is intended as a decision support tool rather than a diagnostic alternative. Its output should be combined with clinical judgment and local antimicrobial management policies. Finally, PPV essentially depends on the prevalence of the outcome; in an environment with a low prevalence of CRKP, clinicians may choose a higher probability threshold to increase PPV, but at the expense of sensitivity. The continuous output of the model allows flexible selection of thresholds based on local priorities.

The nomogram model developed in this study translates complex statistical relationships into an intuitive, user-friendly bedside tool. For patients identified as high-risk, clinicians can initiate empirical antibiotic therapy covering CRKP immediately at the time of specimen collection, prior to the availability of antimicrobial susceptibility results. This approach aligns with evidence underscoring the critical importance of early, appropriate therapy for improving outcomes in CRKP infections. For instance, Mantzarlis et al. (40) demonstrated that early combination regimens (e.g., ceftazidime/avibactam plus aztreonam) are pivotal. Capitalizing on this initial “golden hour” for treatment can substantially reduce the high mortality rates associated with failure of initial empirical therapy (41). Conversely, for low-risk patients, the model could support antimicrobial stewardship efforts by providing objective data to justify avoiding unnecessary broad-spectrum or reserve antibiotics, thereby helping to curb further resistance development (20). The accompanying nomogram and SHAP-based explanations are designed to bridge the gap between complex algorithms and clinical workflow, fostering interpretability and trust.

### 4.4 Study limitations

This study has several limitations. Firstly, the single-center retrospective design may introduce selection bias, and the lack of external validation means the model’s generalizability to other settings, where local epidemiology and clinical practices may differ, remains unproven. While all models were validated on the same internal set (*n* = 120) to ensure fair comparison, this does not substitute for external validation in independent cohorts. Future multicenter studies are essential to assess the model’s transportability and real-world performance. Secondly, while the sample size of 63 CRKP events (44 in the training set) meets the current recommendation of 5–9 events per variable for penalized regression methods such as LASSO, it remains relatively modest. This may limit the ability to adequately capture rare features or rare combinations of risk factors, and the precision of effect estimates is correspondingly constrained. Thirdly, a fixed 90-day window was used to assess antibiotic exposure without distinguishing between short-term and long-term exposures. Although we recognize that the impact of antibiotic selection pressure is likely to be time-dependent - the impact of recent exposure is greater, the heterogeneity of our data sources makes it impossible to systematically extract the exact exposure date. This simplification may weaken the real association between recent antibiotic use and CRKP risk, as our estimates represent the use or non-use over the entire 90-day period. However, the 90-day window is consistent with the clinical guidelines (20) and also reflects the information that clinicians can usually obtain at the bedside. Prospective studies of standardized, date-labeled drug records are needed in the future to better elucidate the time dynamics of antibiotic exposure. Fourthly, this study did not distinguish carbapenemase types (e.g., KPC, NDM, OXA-48). These methods were not routinely performed throughout the retrospective study period. Different types of carbapenemases may have different epidemiological characteristics, risk factors and therapeutic significance, which has certain limitations for clinical significance. However, the main goal of our model is to predict phenotypic resistance - the information that clinicians have the most direct access to when collecting specimens. Future research will combine molecular data to explore whether risk factors can be distinguished according to the type of carbapenemase, and whether predictive models of specific mechanisms can be used to improve clinical practicality. Finally, a modest PPV of 0.567 highlights the risk of false positives that can lead to unnecessary broad-spectrum antibiotic use. This shows that the model is a decision support tool, rather than a clinical judgment standard. Future iterations of the model can incorporate additional predictors. Prospective implementation studies are needed to assess the impact of antibiotic prescribing and patient outcomes in the real world.

## 5 Conclusion

In conclusion, this study demonstrates that a machine learning model combining LASSO feature selection and XGBoost, based exclusively on routine clinical data available at the point of care, can provide a reliable early assessment of CRKP infection risk in ICU patients. The model identifies factors associated with high-risk patients characterized by prior resistance exposure, elevated inflammatory markers, and greater illness severity. With its favorable discrimination, calibration, and focus on interpretability, this tool holds promise for supporting more precise empirical therapy and advancing antimicrobial stewardship in the critical care setting. Future research should focus on external validation and prospective evaluation of its real-world clinical effectiveness and impact.

## Data Availability

The data supporting the findings of this study are available from the corresponding author upon reasonable request.
